# Ischémie aiguë non traumatique du membre inférieur chez un nourrisson de 1 an: cas clinique et revue de la littérature

**DOI:** 10.11604/pamj.2015.21.171.6448

**Published:** 2015-07-02

**Authors:** Rachid Zaghloul, Comlan Mawuko Blitti, Hamid Jiber, Abdellatif Bouarhroum

**Affiliations:** 1 Service de Chirurgie Vasculaire, CHU Hassan II Fès, Maroc

**Keywords:** Ischémie aiguë, membre inférieur, nourrisson

## Abstract

L'ischémie aiguë non traumatique du membre inférieur du nourrisson est une affection rare mais aux conséquences graves. Les étiologies sont multiples, mais il existe des cas idiopathiques. Nous rapportons un cas d'ischémie aiguë idiopathique d'un nourrisson traité médicalement. Malgré le retard de consultation, l’évolution a été spectaculaire avec une régression complète des signes en trois jours.

## Introduction

L'ischémie aiguë du nourrisson est rare mais grave par ses répercussions fonctionnelles et vitales. Il s'agit d'un drame qui engage une course contre la montre; tous les moyens doivent être mis en jeu pour préserver le pronostic fonctionnel du membre. L’étiologie est dominée par les traumatismes artériels liés aux cathétérismes [1]. Les ischémies non traumatiques du membre constituent une entité exceptionnelle. Peu d’études rapportent les ischémies aiguës non traumatique chez les nourrissons [1, 2]. Nous rapportons un cas d'ischémie aigue non traumatique d'un nourrisson et discutons des aspects diagnostique, thérapeutique et évolutif par une revue de la littérature.

## Patient et observation

**Figure 1:**
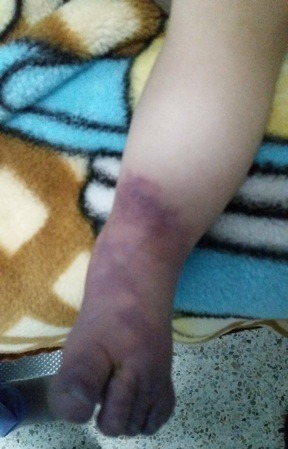
Aspect du pied à l'admission

**Figure 2:**
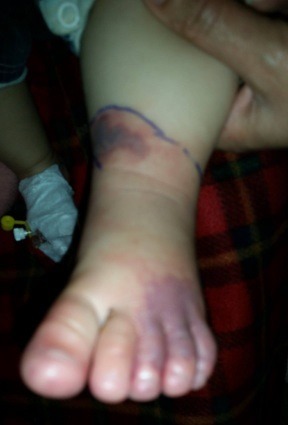
Aspect du dos du pied au troisième jour du traitement

**Figure 3:**
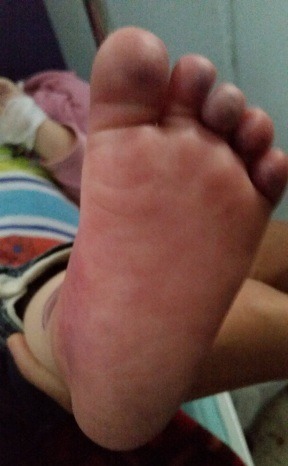
Aspect de la plante du pied au troisième jour du traitement

Il s'agit d'un nourrisson de douze mois, de sexe féminin, sans antécédent pathologique médical personnel et familial, admis pour une cyanose du pied gauche évoluant depuis 18 heures avant l'admission. La maladie a commencé par des cris et pleurs incessants, puis la constatation par les parents d'une cyanose du 2^ème^ orteil des 2 membres. Ces symptômes ont ensuite évolué vers une cyanose du pied gauche alors que les symptômes du membre controlatéral ont régressé. Une consultation aux urgences 18 heures après le début des symptômes a noté: un nourrisson apyrétique, une cyanose du pied gauche arrivant au tiers inférieur de la jambe (Figure 1), une froideur, un œdème du pied, une sensibilité douloureuse du pied, une absence de mobilité des orteils et une abolition des pouls distaux du membre inférieur gauche. L’échographie-doppler a retrouvé une perméabilité des axes artériels jusqu’à la jambe, avec une absence de flux artériel en distal sans visualisation de thrombose. Le bilan biologique réalisé à l'admission a noté: une anémie avec un taux d'hémoglobine à 8,7g/dL, un taux de créatine kinase à 168 UI/L (soit 1,15 fois la normale), un taux de Lactate déshydrogénase à 501,0 UI/L (soit 2 fois la normale), une vitesse de sédimentation à 22,0 mm la première heure et 45,0 mm la deuxième heure, une protéine C réactive à 2,0 mg/L (normal). Le reste de l'examen biologique était normal. Le traitement a consisté en une injection de 100 unités/kg d'héparine non fractionnée en bolus, puis en continue à raison de 20 unités/kg/h ayant permis d'avoir un temps de céphaline activée à 2,4 fois la normale. L’évolution était marquée par une régression progressive de la cyanose (Figure 2 et Figure 3), un réchauffement du membre et une reprise de la mobilité. La sensibilité douloureuse du pied a disparu au 3ème jour du traitement. Le bilan étiologique a consisté en une échographie cardiaque, un bilan de la crase sanguine, un bilan infectieux, un bilan de thrombophilie, qui était normal. L’évolution a été favorable avec un recul de 3 mois.

## Discussion

Parmi les 151 cas d'ischémie aiguë des membres rapportés par Kayssi et al. Sur une période de 13 ans, seulement 14 cas (9%) étaient non traumatique [1]. La littérature de cette affection se résume souvent aux cas cliniques [2]; traduisant ainsi sa rareté. Le diagnostic positif de l'ischémie aigue est clinique. L'imagerie permettant d'infirmer le diagnostic dans un but pré-thérapeutique. L’échographie-doppler est souvent suffisant mais dans certain cas, on peut avoir recours à une artériographie ou un angioscanner [3]. Le diagnostic de notre patiente était patent cliniquement, et a été infirmé par l’échographie-doppler. L'ischémie aigüe de l'enfant est une affection polymorphe. Les étiologies sont multiples, bien dominées par les cathétérismes artériels dans 91 à 92% (1,3). Les orientations étiologiques non traumatiques varient surtout en fonction de la tranche d’âge. En période néonatale, les anomalies génétiques et les problèmes au cours de la grossesse sont suspectés. Une meilleure connaissance actuelle des anomalies constitutionnelles et acquises de l'hémostase, permet de mieux rechercher les étiologies. Ainsi des ischémies aigues de nouveaux nés liées à une hétérozygotie pour le polymorphisme 677C>T de la méthylène-tétrahydrofolate-réductase, et une mutation du gène du facteur V de Leiden [4, 5], ont été rapporté. Aussi, l'administration de certains médicaments comme l'indométacine au cours de la grossesse [6], une déshydratation hypernatrémique [7] pourraient être associé à certaines formes d'ischémies aigues du nouveau né. Chez les enfants d’âge supérieur à 3 ans, les étiologies bien que rare peuvent être une vascularite, un piège poplité, le syndrome des anti-phospholipides, une mutation du gène de la prothrombine, une hyperhomocystéinémie [1, 8].

Daskalaki et al. ont rapporté une ischémie aiguë chez un nourrisson due à une infection au Streptocoque beta-hémolytique du groupe A [2]. Mais la littérature rapporte des cas d'ischémie aigue idiopathique [1], comme dans notre cas. Il n'existe pas de consensus sur la prise en charge des ischémies aiguës chez l'enfant. Mais la capacité qu'a ce groupe particulier de patient à développer rapidement les collatérales artérielles, prône de plus en plus vers une attitude non interventionnelle en première intention même en cas de traumatisme artériel [1, 3]. Le traitement médical consistera en l'héparine non fractionné ou en enoxaparine, en activateur tissulaire du plasminogène, en aspirine ou warfarine en fonction du contexte. En cas, d’échec, d'autres traitement mini-invasif comme le bloc nerveux sympathique pour lever le spasme artériel [9], la thrombolyse in situ ont donné de bons résultats [1]. La chirurgie consiste en une embolectomie ou un pontage le plus souvent, mais nécessite des connaissances en microchirurgie pour assurer la qualité du geste [10]. L’évolution est souvent favorable lorsque le diagnostic est posé tôt, et un traitement institué dans le meilleur délai. Dans notre cas, un délai de 18 heures depuis le début des symptômes a permis une amélioration quasi-totale des symptômes en trois jours de traitement médical.

## Conclusion

L'issue dramatique de l'ischémie aiguë du nourrisson est l'amputation. Tant elle constitue un drame pour le devenir fonctionnel du nourrisson, elle constitue également un traumatisme psychologique majeur aux parents. Tout doit être mis en œuvre pour éviter cette situation. Le traitement doit dans la mesure du possible être médical, même en cas de diagnostic supposé tardif. La chirurgie est réservée aux échecs du traitement médical premier. Une recherche étiologique est indispensable afin d'instaurer un traitement de fond pour éviter les récidives, mais l'on doit garder à l'esprit que les ischémies aigües idiopathiques existent.

## References

[1] Kayssi A, Shaikh F, Roche-Nagle G, Brandao LR, Williams SA, Rubin BB (2014). Management of acute limb ischemia in the pediatric population. J Vasc Surg.

[2] Daskalaki MA, Boeckx WD, DeMey A, Franck D (2013). Toxic shock syndrome due to group A beta-hemolytic streptococcus presenting with purpurafulminans and limb ischemia in a pediatric patient treated with early microsurgical arteriolysis. J Pediatr Surg.

[3] Matos JM, Fajardo A, Dalsing MC, Motaganahalli R, Akingba GA, Murphy MP (2012). Evidence for nonoperative management of acute limb ischemia in infants. J Vasc Surg..

[4] Hakim A, Ben Hamad A, Regaieg R, Gargouri A (2014). [Intrauterine upper limb ischemia due to a heterozygous mutation (677C>T) of the methylene-tetrahydrofolatereductase gene]. Arch PédiatrieOrgane Off SociéteFrPédiatrie.

[5] Khriesat WM, Al-Rimawi HS, Lataifeh IM, Al-Sweedan S, Baqain E (2010). Intrauterine upper limb ischemia associated with fetal thrombophilia: a case report and review of the literature. ActaHaematol..

[6] Arad I, Bar-Oz B, Amit Y, Ergaz Z, Peleg O (1995). Neonatal limb ischemia following maternal indomethacin treatment in twin pregnancies. J Perinat Med..

[7] Dogra S, Agrawal SK, Jindal R, Suri D, Ahluwalia J, Singh S (2011). Peripheral gangrene in a breast fed neonate-is hypernatremic dehydration the cause?. Indian J Pediatr..

[8] Bernheim JW, Hanson J, Hansen J, Faries P, Kilaru S, Winchester P (2004). Acute lower extremity ischemia in a 7-year-old boy: an unusual case of popliteal entrapment syndrome. J Vasc Surg..

[9] De Carolis MP, Bersani I, Piersigilli F, Rubortone SA, Occhipinti F, Lacerenza S (2014). Peripheral nerve blockade and neonatal limb ischemia: our experience and literature review. Clin Appl Thromb Hemost..

[10] Zetlitz E, Weiler-Mithoff E, Turner T (2008). Idiopathic neonatal ischemia in the upper limb: the role of the microsurgeon. Am J Perinatol..

